# Heptamethine carbocyanine dye-mediated near-infrared imaging of canine and human cancers through the HIF-1α/OATPs signaling axis

**DOI:** 10.18632/oncotarget.2464

**Published:** 2014-10-25

**Authors:** Changhong Shi, Jason Boyang Wu, Gina C-Y. Chu, Qinlong Li, Ruoxiang Wang, Caiqin Zhang, Yi Zhang, Hyung L. Kim, Jing Wang, Haiyen E. Zhau, Dongfeng Pan, Leland W.K. Chung

**Affiliations:** ^1^ Laboratory Animal Center, the Fourth Military Medical University, Xi'an, Shaanxi 710032, China; ^2^ Uro-Oncology Research Program, Department of Medicine, Cedars-Sinai Medical Center, Los Angeles, CA 90048, USA; ^3^ Department of Radiology, the University of Virginia, Charlottesville, VA 22908, USA; ^4^ Department of Surgery, Samuel Oschin Comprehensive Cancer Institute, Cedars-Sinai Medical Center, Los Angeles, CA 90048, USA; ^5^ Department of Nuclear Medicine, Xijing Hospital, the Fourth Military Medical University, Xi'an, Shaanxi 710032, China

**Keywords:** HIF-1α, imaging canine cancer, imaging human prostate cancer, near-infrared dye, organic anion-transporting polypeptides

## Abstract

Near-infrared (NIR) fluorescence imaging agents are promising tools for noninvasive cancer imaging. This study explored the specific uptake and retention of a NIR heptamethine carbocyanine MHI-148 dye by canine cancer cells and tissues and human prostate cancer (PCa) specimens and also the dye uptake mechanisms. The accumulation of MHI-148 was detected specifically in canine cancer cells and tissues and freshly harvested human PCa tissues xenografted in mice by NIR fluorescence microscopy and whole-body NIR optical imaging. Specific dye uptake in canine spontaneous tumors was further confirmed by PET imaging. Higher hypoxia-inducible factor-1α (HIF-1α) and organic anion-transporting polypeptide (OATP) protein and mRNA expression was demonstrated by multiplex quantum dots labeling and qPCR in tumors over that of normal tissues. Treating cancer cells with HIF-1α stabilizers activated HIF-1α downstream target genes, induced *OATP* superfamily gene expression and enhanced cellular uptake and retention of NIR dyes. Moreover, silencing *HIF-1α* by siRNA significantly decreased OATP mRNA expression and blocked NIR dye uptake in cancer cells. Together, these results demonstrated the preferential uptake of NIR dyes by canine and human cancer cells and tissues via the HIF-1α/OATPs signaling axis, which provides insights into future application of these dyes for cancer detection and treatment.

## INTRODUCTION

Near-infrared (NIR) heptamethine carbocyanine dyes are a new class of heterocyclic polymethine cyanine compounds with significant advantages for *in vivo* imaging due to their high extinction coefficients and large Stokes' shifts, capable of generating strong fluorescence emission at the range of 700–1000 nm [1–3]. They can be detected with ease with little interference from auto-fluorescence often generated from tissue background [4]. Several of the dyes have become commercially available in recent years, such as Cy5.5 [5] and IRDye800-CW [6], which have been coupled with peptides or antibodies and successfully used for the targeted visualization of neoplastic cancers in animal models. Indocyanine green (ICG), the prototype of this class of NIR dye, has long been used as a contrast agent in clinical imaging [7], and for the diagnosis of liver cirrhosis, liver fibrosis and gastrointestinal vascular defects. Because of poor stability, rapid decomposition in polar solution and lack of tumor targeting specificity, ICG has limited application as a specific imaging agent for cancer [8]. Mechanistically, transmembrane movement of these dyes is thought to be mediated by organic anion-transporting polypeptides (OATPs), cell membrane-bound channels mediating cellular transport of amphiphilic compounds, including drug and other endogenous and exogenous substrates and metabolites. OATPs may play a key role in determining the specific uptake of NIR dyes by cancer cells [9, 10].

We previously found that several new synthetic heptamethine carbocyanines are preferentially taken up and accumulated by malignant cells [11, 12]. We demonstrated that this unique dye subgroup, consisting of IR-783, MHI-148 and PC-1001, were quite stable in body fluids and could be exploited as dual imaging and targeting agents due to their preferential accumulation and retention in cancer cells but not normal cells [11, 13]. In a series of biosafety studies, these dyes were found to generate no systemic toxicity in experimental mice [11]. We also determined that the cancer-specific dye uptake and retention could be antagonized by the competitive OATP inhibitor bromosulfophthalein [11], in agreement with recent evidence indicating that OATP1B3 dominantly controls the transport of heptamethine carbocyanine dyes into cancer cells [14]. There is potentially a regulatory relationship between OATPs and tumor hypoxia, a common condition typically associated with changes in metabolism and found in different cancer types [15–18]. With the molecular basis of cancer-specific uptake and retention characterized, these NIR dyes could potentially be employed as non-invasive dual imaging and targeting agents for canine and human cancers without the need of chemical conjugation to ligands that bind to cell surface receptors.

Given that non-invasive tumor imaging has been reproducible and straightforward with experimental mice, a critical pre-clinical study is to determine whether the imaging could be recapitulated in larger animals in the same fashion. We extended NIR tumor imaging to the detection of canine tumors. Dogs have long been used as model subjects in drug discovery and developmental research due to their many anatomical and physiological similarities to humans [19], and the human and canine genomes share a higher similarity than human and murine genomes [20]. Dogs develop cancers that recapitulate critical characteristics of human malignancies, such as histological appearance, pathophysiologic behavior and therapeutic response [21]. Moreover, canine cancer models capture essential issues of human cancer in a manner not possible with other animals [22]. Specifically important for this project, dogs are the only other animal that may suffer from prostate cancer (PCa), and both canine and human PCa displayed the phenotypes of bone metastasis and castration-resistance [21].

In the present study, we used domestic dogs carrying different types of spontaneous primary and metastatic tumors to evaluate the NIR dyes for dual tumor imaging and targeting capability in large live animals. Comparative examination of cellular uptake of NIR dyes was performed in both tumor and adjacent normal tissues, which was further extended to canine cancer cell lines and xenograft tumors in athymic mice. The molecular mechanisms underlying NIR dye uptake in canine cancer cells were assessed, with a focus on the convergence of hypoxia signaling and *OATP* expression. The results of the study confirm the specificity and bioavailability of NIR dyes for canine cancer imaging, and provide new support for developing NIR dyes further for clinical detection and therapy for both human and canine cancers.

## RESULTS

### Preferential accumulation of NIR fluorescence dye in canine spontaneous tumors

**Figure 1:**
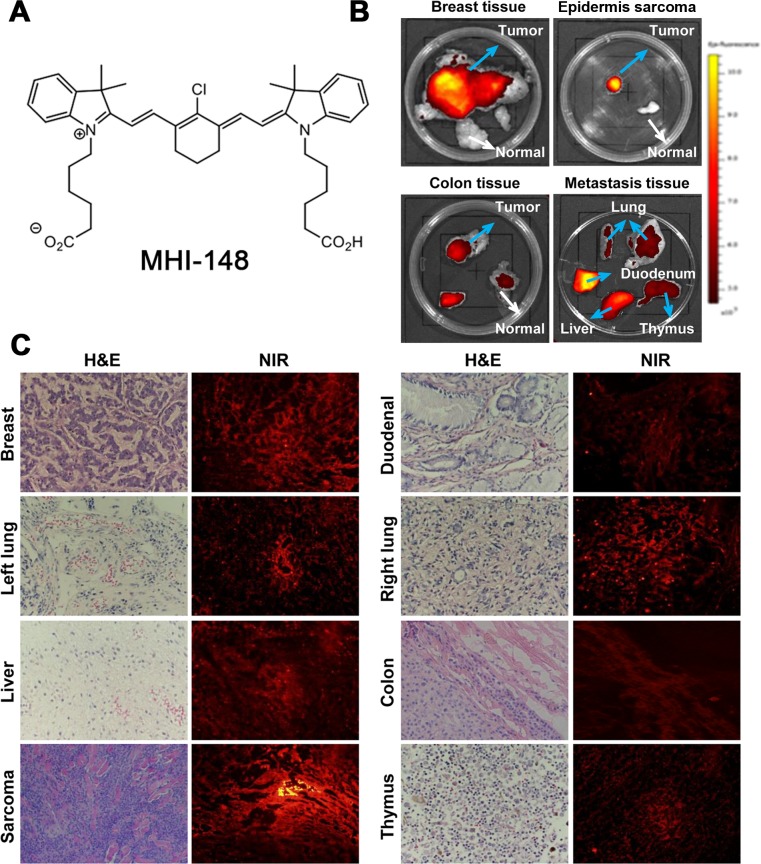
Preferential uptake and retention of MHI-148 dye in canine tumors **(A)** Chemical structure of MHI-148. **(B)** *Ex vivo* NIR fluorescence imaging showed increased uptake of MHI-148 dye by different types of canine spontaneous tumors (blue arrows) as compared to adjacent normal tissues (white arrows). **(C)** Subsequent to *ex vivo* NIR imaging, the tumors were sectioned and subjected to histological (H&E) and NIR fluorescence microscopic analysis (NIR). Intense NIR signals in tumor sections are shown. All the images were at 100× magnification.

Dogs that carried different spontaneous tumors were injected with MHI-148 dye (chemical structure shown in Figure 1A) via femoral vein. Three days later, tumors were removed and subjected to *ex vivo* NIR fluorescence optical imaging. By combining histologic and fluorescence microscopic analyses, we detected strong NIR fluorescence signals taken up by primary and metastatic canine tumor tissues, including breast cancer, liver cancer, lung cancer, duodenal cancer, colon cancer and epidermis sarcoma, while no signal accumulation was observed in adjacent normal tissues (Figure 1B). Tumor histology and the specific NIR dye uptake were further confirmed by H&E staining and fluorescence microscopic analysis of frozen sections, respectively (Figure 1C). The results from these studies demonstrated that, similar to the findings with mice, this unique class of heptamethine carbocyanine dyes can detect spontaneous tumors in large animals.

**Figure 2:**
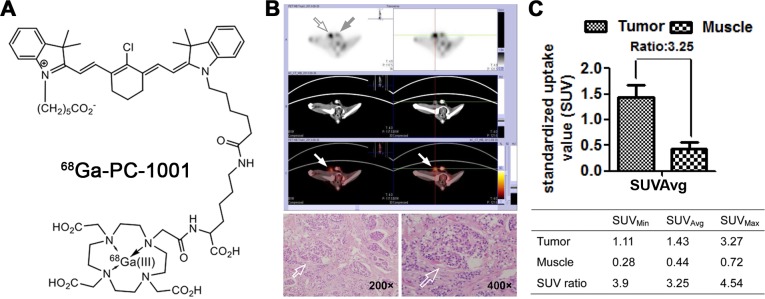
Preferential uptake and retention of ^68^Ga-PC-1001 in canine testicular tumors **(A)** Chemical structure of ^68^Ga-PC-1001. **(B)** The ^68^Ga-PC-1001/PET conjugate was used to detect spontaneous canine testicular tumors. With little signal seen in the right normal testis (gray arrow), this non-invasive imaging detected specific signals in the left inflicted testis (open arrow), which was confirmed by H&E staining (bottom panels; magnification, 200× and 400×). **(C)** From the non-invasive testicular tumor detection, the standardized uptake value (SUV) as well as tumor SUV-to-muscle SUV ratios (T/M) are shown.

To further improve the sensitivity and clinical utility of NIR dyes for deep-tissue tumor detection, we modified the NIR dye by conjugating it with a positron-emitting radionuclide and tested its feasibility for detection of canine testicular cancer. PET/CT scans were performed one hour after intravenous injection of ^68^Ga-PC-1001 (chemical structure shown in Figure 2A) into a dog diagnosed with testicular cancer. The uptake of ^68^Ga-PC-1001 in the testicular cancerous region was clearly enhanced after a short period (Figure 2B, top panels), and the targeted cancerous area was later confirmed by histology (Figure 2B, bottom panels). Moreover, we analyzed the dynamic PET imaging indices including the average, minimal and maximal standardized uptake value (SUV) as well as the tumor SUV-to-muscle SUV ratios (T/M). A T/M ratio of 3.25 for average SUV was demonstrated, indicating effective cancer-specific uptake of the radiolabeled NIR conjugate captured by PET/CT (Figure 2C). Although PET/CT imaging of other deep-tissue tumors has not yet been conducted, this initial study provided proof-of-principle results for non-invasive tumor detection in domestic dogs.

### Uptake of NIR fluorescence dye by canine cancer cells and tumor xenografts

**Figure 3:**
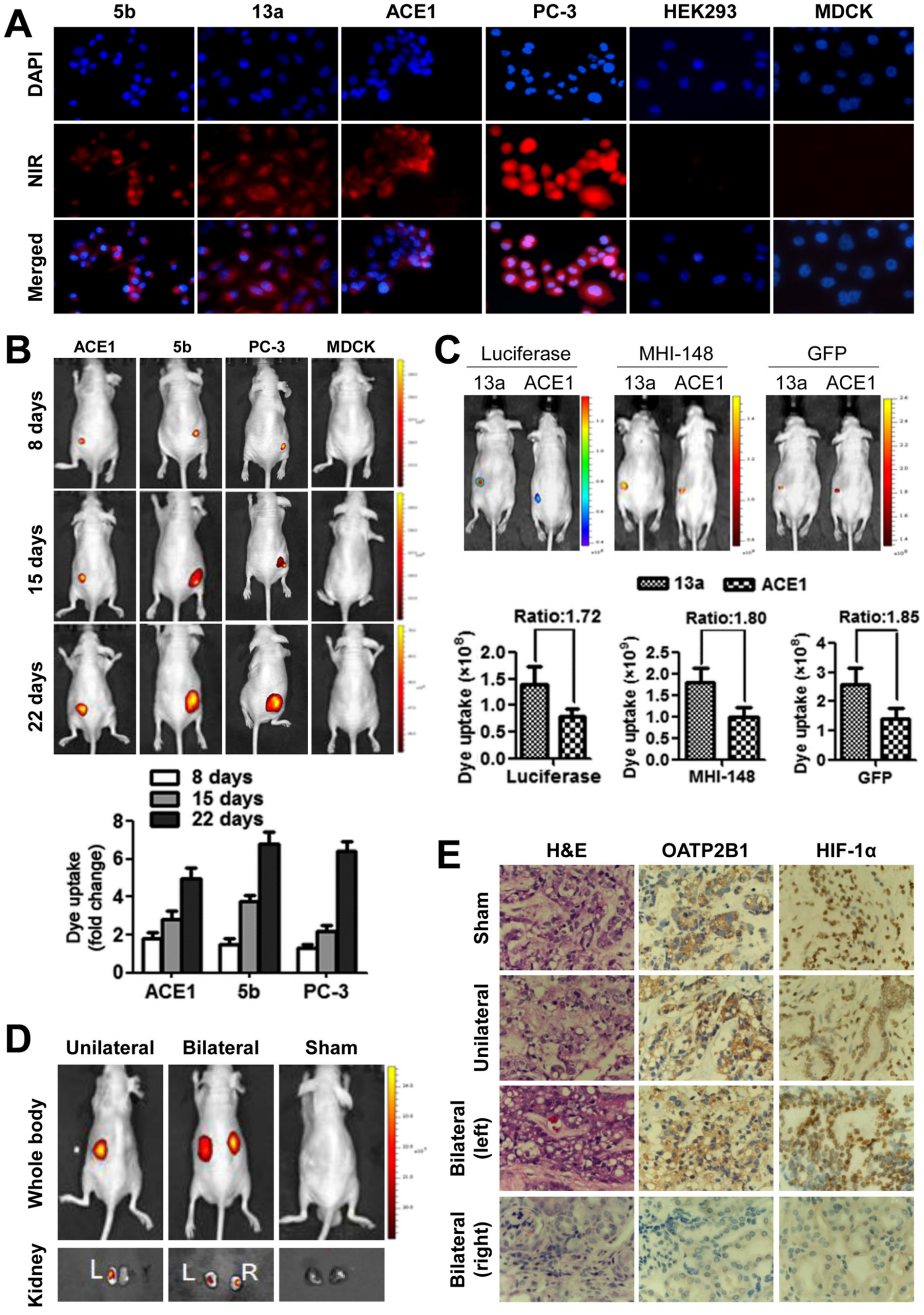
Specific uptake of MHI-148 dye by canine cancer but not normal cells **(A)** Representative NIR fluorescence microscopic images show the significant uptake of MHI-148 dye by canine 5b, 13a and ACE1 and human PC-3 cancer cells but not by normal canine MDCK and human HEK293 cells (magnification, 200×). **(B)** Continued documentation revealed preferential uptake and retention of MHI-148 dye in canine tumor xenografts. Top panels: nude mice bearing subcutaneous xenograft tumors were subjected to NIR fluorescence optical imaging consecutively on days 8, 15, and 22 after implantation. Bottom panels: a histogram summarizes signal intensity detected from xenografts (N=3) at each measurement time point. **(C)** Mice bearing dually luciferase- and GFP-labeled canine cancer cells were subjected to whole-body bioluminescence (Luciferase), NIR (MHI-148) and GFP fluorescence imaging. Signal intensity ratio of canine 13a to ACE1 tumor xenografts with different labels is shown. **(D)** Uptake of the NIR dye by human clinical prostate tumor xenografts implanted under the subrenal capsule of nude mice. **(E)** Examination of dissected xenografts for HIF-1α and OATP2B1 expression following IHC staining (magnification, 200×).

Using different established canine cancer cell lines as models, we sought to determine the *in vitro* uptake of NIR dye by cancer cells. Canine CHMp-5b and CHMp-13a breast cancer cells and ACE1 PCa cells specifically retained the dye, as detected with strong fluorescence signals by NIR microscopy in comparison with normal canine MDCK renal cells which yielded background signal (Figure 3A). In addition, human PC-3 PCa cells and HEK293 normal kidney cells were used in parallel as positive and negative controls, respectively, as these cells display differential NIR uptake and retention [11, 13].

To determine whether the characteristic tumor imaging could be recapitulated *in vivo* with tumor xenografts, we injected dually luciferase- and green fluorescence protein (GFP)-tagged 5b and ACE1 cells (1 × 10^6^ cells/site) subcutaneously into nude mice to allow xenograft tumor formation. The tumor-bearing animals were then subjected to NIR tumor detection at different time points subsequent to tumor implantation. Strong NIR fluorescence signals were clearly captured from the injection sites eight days after tumor cell injection (Figure 3B, top panels). The intensity of NIR signals centered at the tumor region demonstrated an increasing trend over three time points of measurement (days 8, 15 and 22) along with tumor growth (Figure 3B, histogram). By contrast, we did not observe either the formation or uptake of NIR dye by MDCK cells injected in parallel (Figure 3B). These results in aggregate demonstrated the tumor-specific targeting ability of the NIR dye regardless of the species.

To compare simultaneously the specificity and consistency of NIR fluorescence imaging with other established imaging approaches, we subjected mice that carried dually luciferase- and GFP-tagged canine 13a and ACE1 tumor xenografts to NIR/GFP fluorescence and bioluminescence imaging. We observed comparable specificity and consistency of signal readout for tumor recognition across different imaging modalities, and moreover, the signal intensity was correlated consistently with tumor size (Figure 3C).

### Uptake of the NIR dye by fresh human prostate cancer specimens implanted in mice

To determine the uptake of NIR dye by human clinical cancer specimens, we implanted freshly harvested human prostate tumor samples into nude mice by subrenal capsular grafting, which allows the tumor tissue to be perfused by the rich blood supply from the site of renal capsule and to survive transiently in mice. Forty-eight hours after the surgery, mice were subjected to whole-body NIR fluorescence optical imaging using our established protocol [12]. NIR signals were detected in xenografts implanted either on one side or both sides of the mice with strong signal/background ratios (Figure 3D). Tumor samples were dissected and further validated by histopathologic examination (H&E stain). Since NIR dye uptake was mediated by OATP channel proteins whose expression was regulated by HIF-1α, which in turn was controlled by hypoxia, a common known pathologic condition in tumor growth, we detected HIF-1α and OATP2B1 protein expression in prostate tumor tissue xenografts but not normal tissues by immunohistochemical (IHC) staining (Figure 3E), suggesting similar mechanisms may be responsible for NIR dye uptake by freshly harvested human PCa tissues and cultured cells.

### Expression of HIF-1α and specific OATPs in canine and human cancer tissues

**Figure 4:**
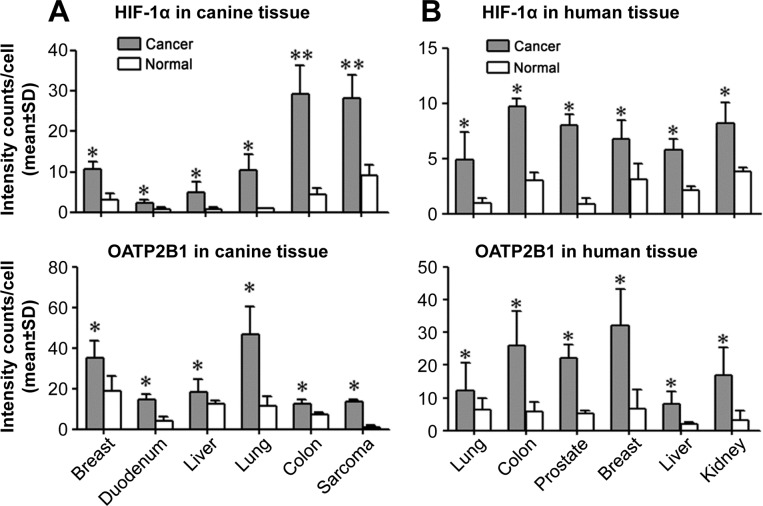
Aberrant expression of *HIF-1α* and selected *OATP* genes in cancers Double QDL analysis was used to quantify HIF-1α and OATP2B1 protein expression in paired cancer and normal (derived from cancer-adjacent normal tissue from the same patient) tissue specimens from either canine **(A)** or human **(B)** cancer patients (N=5 patients for each type of cancer). Average signal intensity counts from 1,000 cells were quantified using inForm software. *P<0.05, **P<0.01.

**Figure 5:**
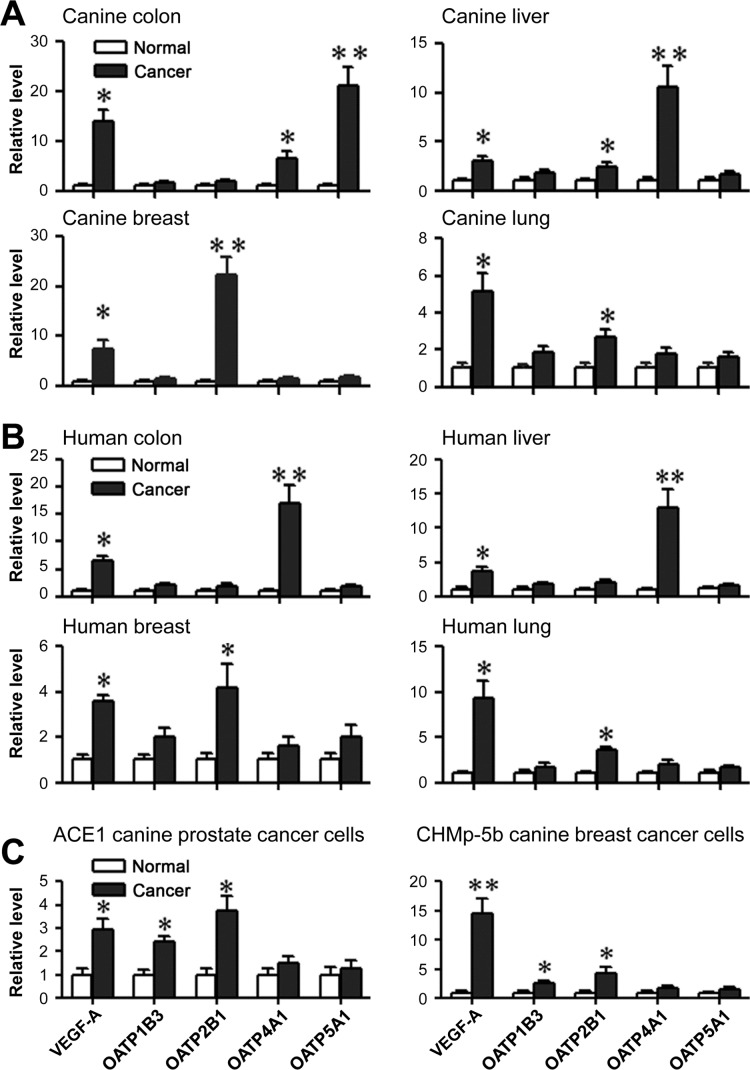
Expression of specific *OATP* genes and HIF-1α-target genes in tumor tissues and cells qPCR analysis of *VEGF-A* and specific *OATP* gene expression in canine cancer tissues **(A)** human cancer tissues **(B)** and canine cancer cell lines **(C)**. Cancer-adjacent normal tissues derived from the same canine (A) or human (B) patients were used as normal controls for respective cancer tissues (N=5 patients for each type of cancer). Primary cultures of normal canine prostate and mammary gland epithelial cells were used as normal controls for ACE1 and CHMp-5b cells, respectively (C). Either human *GAPDH* or canine *Gapdh* was used as an internal control for normalization. Data represent the mean ± SD from three independent experiments. *P<0.05, **P<0.01.

In line with the observation of high levels of HIF-1α and OATP staining in human PCa samples, we surveyed HIF-1α and OATP2B1 protein expression in multiple types of paired cancer-adjacent normal and cancerous canine (breast, duodenal, liver, lung, colon and epidermis sarcoma) and human (lung, colon, prostate, breast, liver and kidney) tissues by double quantum dots labeling (QDL) analysis. As shown in Figure 4 and Supplemental Figure 2, the protein expression of both HIF-1α and OATP2B1 on a per cell basis was significantly higher in tumors than in normal tissues in both canine (Figure 4A) and human (Figure 4B) specimens. Moreover, higher mRNA expression of *VEGF-A* (breast, colon, liver and lung), a HIF-1α-target gene, and select *OATP*s including *OATP2B1* (breast, liver and lung) and *OATP4A1* (colon and liver) were seen in canine cancer tissues relative to cancer-adjacent normal tissues as determined by qPCR (Figure 5A). Similarly, these genes showed increased expression in the same cancer types as compared to respective cancer-adjacent normal tissues in humans (Figure 5B). qPCR analysis further revealed higher mRNA expression of *VEGF-A*, *OATP1B3* and *OATP2B1* in canine ACE1 PCa and CHMp-5b breast cancer cells compared to normal controls. Primary cultures of normal canine prostate and mammary gland epithelial cells were used as normal controls for ACE1 and CHMp-5b cells, respectively (Figure 5C). Together, these results suggest that the specific and higher uptake and accumulation of NIR dye by canine and human cancer tissues could be mediated by hypoxia-induced HIF-1α and specific OATPs.

### HIF-1α regulation of OATPs expression

**Figure 6:**
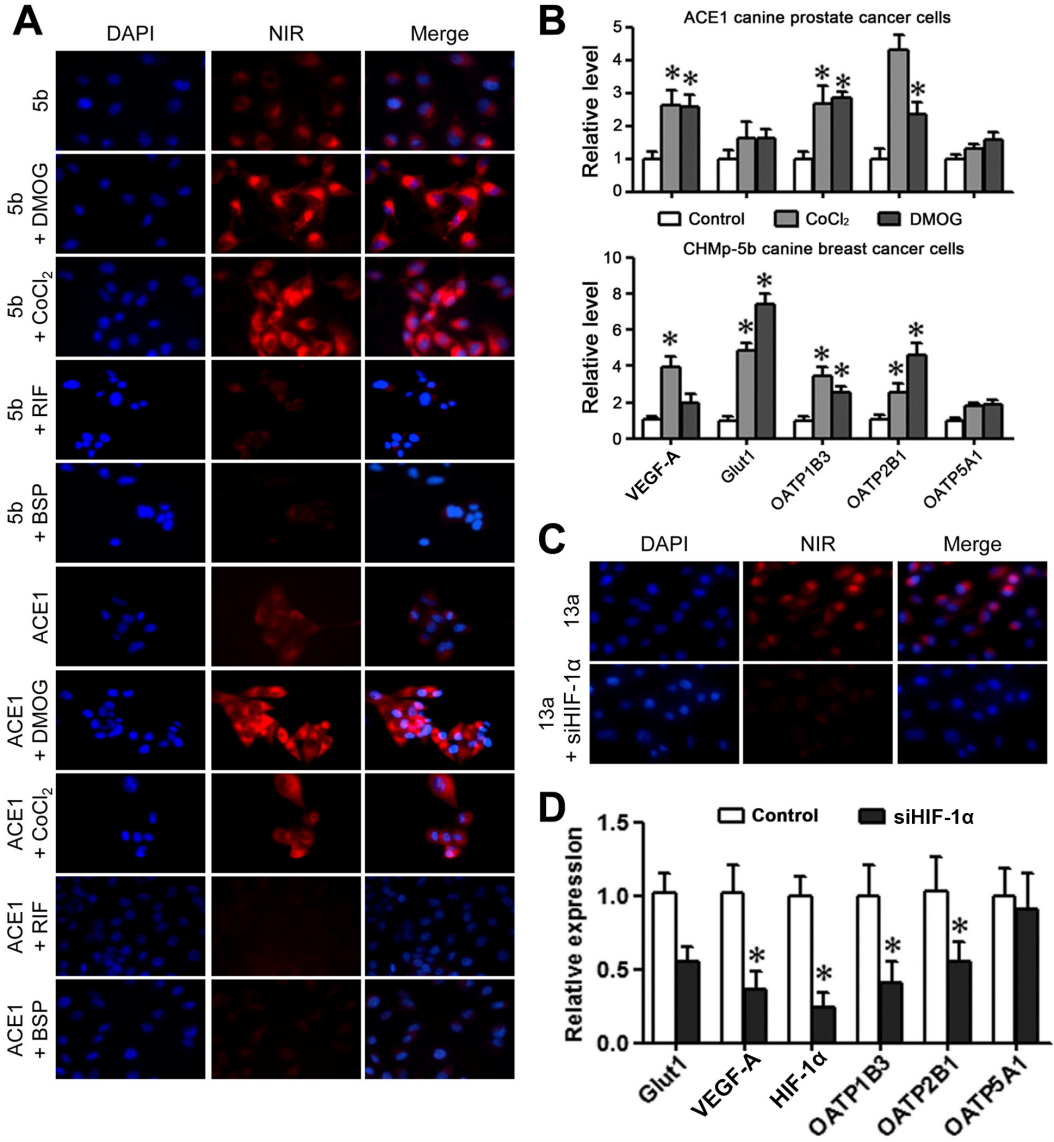
Uptake of the MHI-148 dye through the activation of HIF-1α/OATPs signaling axis **(A)** Uptake of NIR dye by canine cancer cells was regulated by HIF-1α. Canine cancer cells pre-treated with HIF-1α stabilizer DMOG or cobalt chloride showed increased uptake of NIR dye. Canine cancer cells pre-treated with OATP inhibitor RIF or BSP showed reduced NIR dye uptake. All the images are shown at 400× magnification. **(B)** ACE1 and 5b cells were subjected to qPCR analysis of *VEGF-A*, *Glut1*, and selected *OATPs* following treatment with HIF-1α stabilizer DMOG or cobalt chloride. Data represent the mean ± SD from three independent experiments. *P<0.05. **(C)** Reduced uptake of NIR dye following *HIF-1α* knockdown in canine 13a cancer cells as demonstrated by NIR fluorescence microscopy (magnification, 400×). **(D)** qPCR analysis showed reduced expression of *VEGF-A*, *Glut1* and selected *OATP*s following *HIF-1α* knockdown in canine 13a cancer cells. Data represent the mean ± SD from three independent experiments. *P<0.05.

To determine whether the specific uptake and accumulation of NIR dye in canine cancer cells is mediated by HIF-1α, the ACE1 and CHMp-5b cells were pre-treated with HIF-1α stabilizer dimethyloxaloylglycine (DMOG) or cobalt chloride prior to exposure to NIR dye. Both stabilizers significantly enhanced the dye uptake in cells (Figure 6A). Conversely, OATP inhibitors, rifampicin (RIF) and bromosulfophthalein (BSP), attenuated the uptake and retention of NIR dye by cancer cells (Figure 6A). Moreover, we detected significantly enhanced expression of HIF-1α-target genes including *VEGF-A* and *Glut1* in response to DMOG or cobalt chloride treatment, indicating an increase in HIF-1α activity (Figure 6B). Importantly, increased HIF-1α activity was concomitant with the up-regulation of *OATP1B3* and *OATP2B1* mRNA expression, suggesting that HIF-1α could modulate the dye uptake through the induction of *OATP* genes.

To study whether HIF-1α could directly regulate the NIR dye uptake in canine cancer cells, we treated the cells with a *HIF-1α*-targeting siRNA (siHIF-1α) against canine *HIF-1α* gene (Figure 6C). Effective knockdown of *HIF-1α* expression, as determined by reduced *VEGF-A* and *Glut1* mRNA levels, resulted in significant decrease in NIR dye uptake and retention by canine cancer cells under hypoxia (Figure 6D). Furthermore, decreased mRNA expression of both *OATP1B3* and *OATP2B1* genes was demonstrated in the absence of HIF-1α, suggesting a positive regulatory link between HIF-1α and specific OATP genes. These results showed that HIF-1α is capable of directly regulating the expression of select forms of OATPs. This finding reinforced the HIF-1α/OATPs signaling axis as a regulatory node for NIR dye uptake in cancer cells.

## DISCUSSION

Conventional tumor imaging approaches require chemical conjugation of imaging probes with a cancer-specific targeting module of metabolic substrates [23], cell-surface peptides [5], growth factors [24], antibodies [25] and cancer-specific cell-surface biomarkers [26]. However, only limited specific types of cancer cells can be detected by the current modalities, due in large part to tumor cell heterogeneity and the plasticity of gene expression during cancer progression. Additionally, chemical conjugation may alter the active moieties of the imaging probe or the tumor-targeting module, leading to loss of imaging sensitivity or specificity [27].

In this study, we demonstrated that a class of heptamethine carbocyanine dyes, in their native form without chemical conjugation, could be taken up and accumulated specifically in both canine spontaneous tumors and human PCa xenografts (Figures 1 and 3). Since domestic dogs develop spontaneous primary and metastatic tumors in a manner similar to humans, this study provides new data for evaluating MHI-148, a prototype of NIR dye, for its clinical utility as well as understanding its mechanisms of uptake and retention in tumor cells.

Nuclear imaging is an attractive modality for tumor detection because it is non-invasive and quantitative, and can provide dynamic real-time data allowing the diagnosis and follow-up of patients undergoing therapy [13, 28]. The ^18^F-FDG probe has been used extensively in cancer detection and therapeutic monitoring by PET [29, 30]. However, ^18^F-FDG showed short half-life, low spatial resolution, and abundant uptake in brain tissues due to its high basal metabolic rates. An alternative strategy is to develop a dual NIR fluorescence-radioisotope imaging approach [31]. In line with this need, a dual NIR-PET agent integrating ICG with a radioisotope (^99^mTc) and nanoparticles for PCa was recently reported. After injecting this agent prior to surgery, it was observed that this dual NIR-PET approach provided improved surgical guidance via multimodality imaging [32]. Since ICG has no tumor-seeking properties, it is highly likely that the tumor-homing NIR dye approach as described here could provide even greater advantages for recognizing tumor margins at the time of surgery.

In the present study, we synthesized a ^68^Ga-PC-1001 PET/NIR probe by conjugating the NIR dye, PC-1001, with a DOTA chelator and subsequently chelating with ^68^Ga for independent PET and fluorescence imaging. We previously demonstrated that the DOTA moiety did not affect the dye uptake by cancer cells *in vitro* [13]. This two-component probe is the first example of a novel cancer-specific fluorescence dye with both targeting and detection properties. ^68^Ga-PC-1001 was accumulated preferentially in canine cancer tissues over normal tissues (Figure 2), further indicating that the preferential uptake and retention of NIR dyes by cancer cells was independent of imaging modalities. This dual NIR fluorescence and radioactive imaging modality has the merits of simplicity, convenience and high throughput. It optimizes the parameters in a simplified manner and could be easily validated prior to final live animal and human PET imaging of primary and metastatic tumors.

Consistent with tissue-specific expression of OATPs [33], we conducted microarray analysis and confirmed by multiplex QDL (for protein expression) and qPCR (for mRNA expression) that specific *OATP* genes could mediate cancer-specific NIR dye uptake. We found high expression of select *OATP*s mRNA, including *OATP1B3*, *OATP2B1*, *OATP4A1* and *OATP5A1*, in canine cancer cells and tissues but not normal controls (Figure 5). Increased expression of these *OATP* genes in cancer cells may directly promote the cellular uptake and retention of NIR dye. This concept is supported by our observations that pharmacological inhibition of OATP activities by competitive inhibition blocked the dye uptake and retention (Figure 6).

In the presence of oxygen HIF-1α protein is subjected to rapid degradation [34], which may contribute to the relatively low levels of OATPs in normal tissues, leading to a relatively inactive uptake and retention of NIR dye. In cancer cells, the corresponding mechanisms associated with NIR dye uptake could involve the aberrant activation of *HIF-1α* and its transcription factor activity, resulting in direct induction of *OATP*s through binding to hypoxia-response elements within *OATP* promoters. Both HIF-1α and OATP were found to be highly expressed in canine and human cancer tissues in comparison with adjacent normal tissues (Figure 4). The regulatory relationship between hypoxia/HIF-1α and OATPs was confirmed by our observations that treatment of canine cancer cells with HIF-1α stabilizers induced the expression of select *OATP* genes in addition to known HIF-1α-target genes [35, 36], which was further correlated with increased uptake of NIR dye (Figure 6). In contrast, knockdown of *HIF-1α* in canine cancer cells reduced the expression of *OATP1B3* and *OATP2B1* along with HIF-1α-responsive genes, which was concurrent with reduced uptake of NIR dye. This study thus functionally linked OATPs with hypoxia/HIF-1α signaling in the canine cancer model system, providing mechanistic insights for NIR fluorescence tumor imaging.

In summary, we demonstrated the specific accumulation of NIR heptamethine carbocyanine dyes in canine cancer cells, cancer tissues harvested from the spontaneous primary and metastatic tumors of domestic dogs, and human PCa tissues implanted under the mouse kidney capsule. The uptake and retention of NIR dye by cancer but not normal tissues was further confirmed in canine testicular cancer using ^68^Ga-PC-1001 as the radioactive probe for PET/CT scan. We assessed the mechanisms of NIR dye uptake in canine and human cancer cells and tissues and demonstrated that NIR dye uptake and retention in cancer but not normal cells and tissues was likely mediated through the HIF-1α/OATPs signaling axis. Our results establish the basis for developing this class of NIR dyes from bench to bedside with the prospect of improving cancer diagnosis, prognosis and treatment.

## METHODS

### Cell lines and reagents

The canine CHMp-5b (5b) and CHMp-13a (13a) breast cancer cell lines [37] were kindly provided by Dr. Takayuki Nakagawa (The University of Tokyo, Tokyo, Japan) and cultured in RPMI-1640 medium containing 10% fetal bovine serum (FBS), 5 mg/L gentamicin sulfate and 6 mg/L fungizone. The canine ACE1 PCa cell line [38, 39] was kindly provided by Dr. Tom Rosol (The Ohio State University, Columbus, OH) and cultured in RPMI-1640 medium containing 10% FBS, penicillin (100 U/ml) and streptomycin (100 μg/ml). Normal canine renal MDCK, normal human embryonic kidney HEK293 and human PC-3 PCa cell lines were obtained from the American Type Culture Collection (ATCC, Manassas, VA) and cultured in RPMI-1640 medium containing 10% FBS, penicillin (100 U/ml) and streptomycin (100 μg/ml). 4′,6-diamidino-2-phenylindole (DAPI), cobalt chloride, rifampicin (RIF) and bromosulfophthalein (BSP) were purchased from Sigma-Aldrich (St. Louis, MO). Dimethyloxaloylglycine (DMOG) was purchased from Millipore (Billerica, MA). Rabbit polyclonal anti-HIF-1α and anti-OATP2B1 antibodies both predicted to react with canine species were purchased from Novus Biologicals (Littleton, CO).

### Synthesis of heptamethine carbocyanine dye

The heptamethine carbocyanine MHI-148 and PC-1001 dyes were synthesized and purified as described previously [11, 13]. These dyes were dissolved in dimethyl sulfoxide (DMSO), filtered through a 0.22 μm filter, and stored at 4 ^o^C before use.

### Radiolabeling of heptamethine carbocyanine PC-1001 dye

Radiolabeling was carried out by incubating ^68^Ga in 400 μl of NH_4_Ac (0.1 N, pH 5.5) with 50 μg of PC-1001 in 200 ml of methanol at 40 ^o^C for 30 minutes. The reaction mixture was purified with HPLC using ^18^C semi-preparative columns with a non-gradient buffer of 80% acetonitrile and 0.02% trifluoric acid. The HPLC collection was pooled together and the acetonitrile was blown dry by nitrogen gas. The water solution was loaded into a Sep-Pak light ^18^C reverse phase cartridge and ^68^Ga-PC-1001 was eluted with 2 ml of methanol. Finally, the ^68^Ga-PC-1001 residue was dissolved in phosphate buffered saline (PBS).

### Animal studies

Five dogs bearing different spontaneous primary or metastatic tumors were diagnosed by X-ray radiography and tissue biopsy. The information on these dogs is summarized in Supplemental Table 1 and Supplemental Figure 1. All dogs were obtained from the Laboratory Animal Center, the Fourth Military Medical University (FMMU, Xi'an, Shaanxi, China). All dog experiments were carried out following the protocols reviewed and approved by the Institutional Animal Care and Use Committee (IACUC) at the FMMU. Dogs were anaesthetized with intravenous ketamine (5 mg/kg) and diazepam (0.25 mg/kg), and maintained under isoflurane during imaging.

Male 4- to 6-week-old athymic nude mice were purchased from Taconic (Oxnard, CA), housed in the animal research facility at Cedars-Sinai Medical Center (CSMC, Los Angeles, CA), and fed a normal diet and water ad libitum. All mouse studies were performed according to a protocol approved by the IACUC at CSMC.

For determining the uptake of MHI-148 dye in dogs bearing spontaneous tumors, three days before the operation the dogs were injected with MHI-148 (1.5 μmol/kg) through the femoral vein. Tumor tissues after surgical removal were subjected to *ex vivo* NIR optical fluorescence imaging using an IVIS Lumina XR Image System (PerkinElmer, Waltham, MA). Part of each tumor tissue was fixed in 10% formalin. The remainder was used for the preparation of frozen sections and stained with hematoxylin and eosin (H&E) to confirm tumor histology. Frozen tumor sections were further subjected to NIR fluorescence microscopy (Olympus 1×71; Olympus, Melville, NY) with a 75 W Xenon lamp and an indo-cyanine green filter cube (excitation/emission: 750–800/820–860 nm).

For determining the uptake and accumulation of MHI-148 dye in human PCa xenografts in athymic mice, fresh clinical tumor specimens (~0.5 × 0.5 × 0.5 cm^3^) obtained from CSMC were implanted in male nude mice by subrenal capsule grafting. Use of human tissue specimens in research was approved by the institutional review board (IRB) of CSMC. Two weeks later, the mice were injected intraperitoneally with MHI-148 (50 nmol/mouse). After another 48 hours, the animals were subjected to whole-body NIR fluorescence optical imaging. Mice were monitored for an additional 4 weeks prior to euthanasia and necropsy, at which time tumor tissues were harvested for histological analysis.

For imaging canine spontaneous tumors with radiolabeled PC-1001, dogs diagnosed with testicular cancer received a 60-min dynamic PET/CT scan of the pelvis immediately after intravenous injection of ^68^Ga-PC-1001 (5 mCi/animal).

For determining tumor growth by bioluminescence imaging (BLI), mice implanted with luciferase-tagged canine cancer cells were injected intraperitoneally with D-luciferin (3 mg/mouse) (Gold Biotechnology, St. Louis, MO) and subjected to BLI after a 15-minute period.

### Uptake and accumulation of NIR dye in canine cancer cells

Cells were seeded in 24-well plates at the density of 1 × 10^4^ cells/well and incubated for 24 hours, and were then exposed to MHI-148 dye (20 μM) at 37 ^o^C for 30 minutes. The cells were washed twice with PBS to remove free dye before being fixed with cold formalin. Fixed cells were washed twice with PBS and counterstained with DAPI.

For determining the effects of HIF-1α stabilizers and OATP inhibitors on NIR dye uptake, canine cancer cells were pre-incubated with DMOG (1 mM) or cobalt chloride (200 μM) for 24 hours; or with OATP inhibitor RIF (25 μM) or BSP (250 μM) for 30 minutes prior to MHI-148 staining and NIR fluorescence detection. For determining the mediating effect of HIF-1α on NIR dye uptake in canine cancer cells, cells were transiently transfected with a *HIF-1α*-targeting siRNA as described below. Twenty-four hours later, cells were treated with hypoxia (1% O_2_) for another 24 hours prior to MHI-148 exposure and NIR fluorescence detection.

### siRNA knockdown

Small interfering RNA (siRNA) was transiently transfected into cells with Lipofectamine 2000 (Life Technologies, Grand Island, NY) following the manufacturer's instructions. The sequence to silence the translation of canine *HIF-1α* was 5′-CUUAUAUCCCAAUGGAUGAUG-3′ that maps the sequences used for effectively silencing human *HIF-1α* [40]. A non-silencing RNA of 5′-UUCUCCGAACGUGUCACGUUU-3′ was used as a control [41, 42].

### Determination of the expression of HIF-1α and OATPs in canine and human cancer cells

Human cancer specimens used for IHC or qPCR analysis, including lung cancer, PCa, breast cancer, liver cancer, colon cancer and kidney cancer, were obtained from the Xijing Hospital (Xi'an, Shaanxi, China). Use of human tissue specimens in research was approved by the IRB of FMMU. The IHC staining protocol was modified for double QDL as described previously [43, 44]. Briefly, a single tissue section after streptavidin blocking was sequentially incubated with primary antibodies against either HIF-1α and OATP2B1. Biotinylated secondary antibody was used and detected by streptavidin-conjugated quantum dot at the specified wavelength. For parallel control staining, PBS was used to replace primary antibodies to the immediate adjacent sections. Quantification of each double QDL image by determining the signal intensity of each protein was conducted by image acquisition and deconvolution using Nuance 3.0.2 and inForms 1.4.0 software.

Total RNA from fresh tissue and cell samples was isolated using an RNeasy Mini Kit (Qiagen, Valencia, CA). Total RNA in canine and human cancer tissues were extracted and purified from formalin-fixed paraffin-embedded (FFPE) tumor sections by an RNeasy FFPE kit (Qiagen). For qRT-PCR analysis, RNA samples were reverse-transcribed to cDNA using M-MLV reverse transcriptase (Promege, Madison, WI) as described previously [45]. The transcript levels of select *OTAP* genes and HIF-1α-target genes including *VEGF-A* and *Glut1* were determined in both canine and human cancer tissues. PCR reaction was conducted using SYBR Green PCR Master Mix on an Applied Biosystems 7500 Fast Real-Time PCR system (Applied Biosystems, Carlsbad, CA). Sequences of primers for qPCR are summarized in Supplemental Table 2.

### Statistical analysis

Data represent the mean ± SD from at least three independent experiments. The statistical significance of all data was analyzed by two-tailed unpaired Student's *t* test, and a *P* value less than 0.05 was considered as statistically significant.

## SUPPLEMENTARY MATERIAL FIGURES AND TABLES


