# Supplementary material for: Heptamethine carbocyanine dye-mediated near-infrared imaging of canine and human cancers through the HIF-1α/OATPs signaling axis
Source: Oncotarget. 2014 Oct 25;5(20):10114–26. doi: 10.18632/oncotarget.2464 (PMC4259409; doi:10.18632/oncotarget.2464)
Supplement: Supplementary file 1 [file oncotarget-05-10114-s001.pdf]

SUPPLEMENTARY FIGURES AND TABLES

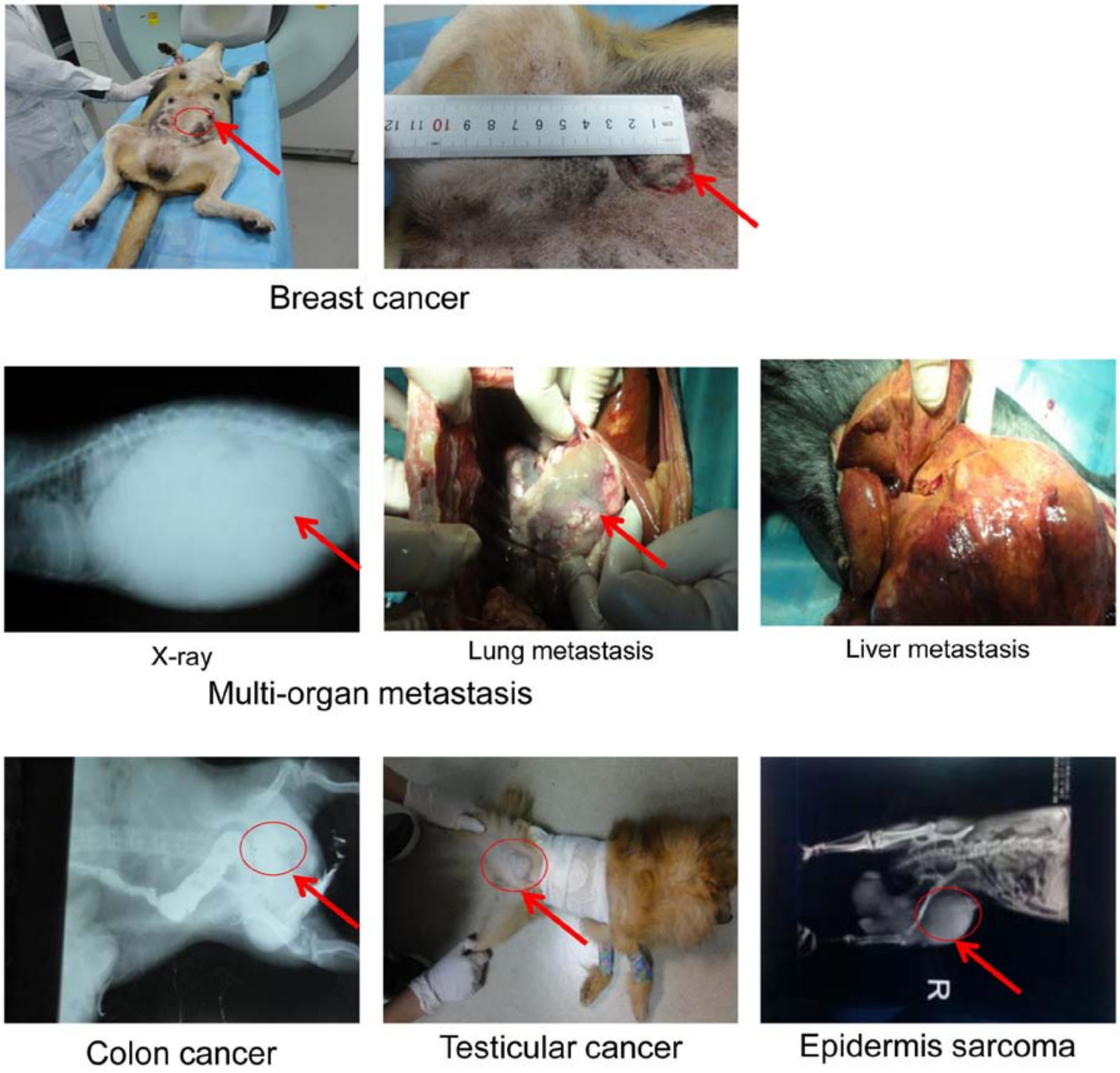

Supplementary Figure S1: Images of dogs bearing different spontaneous tumors.

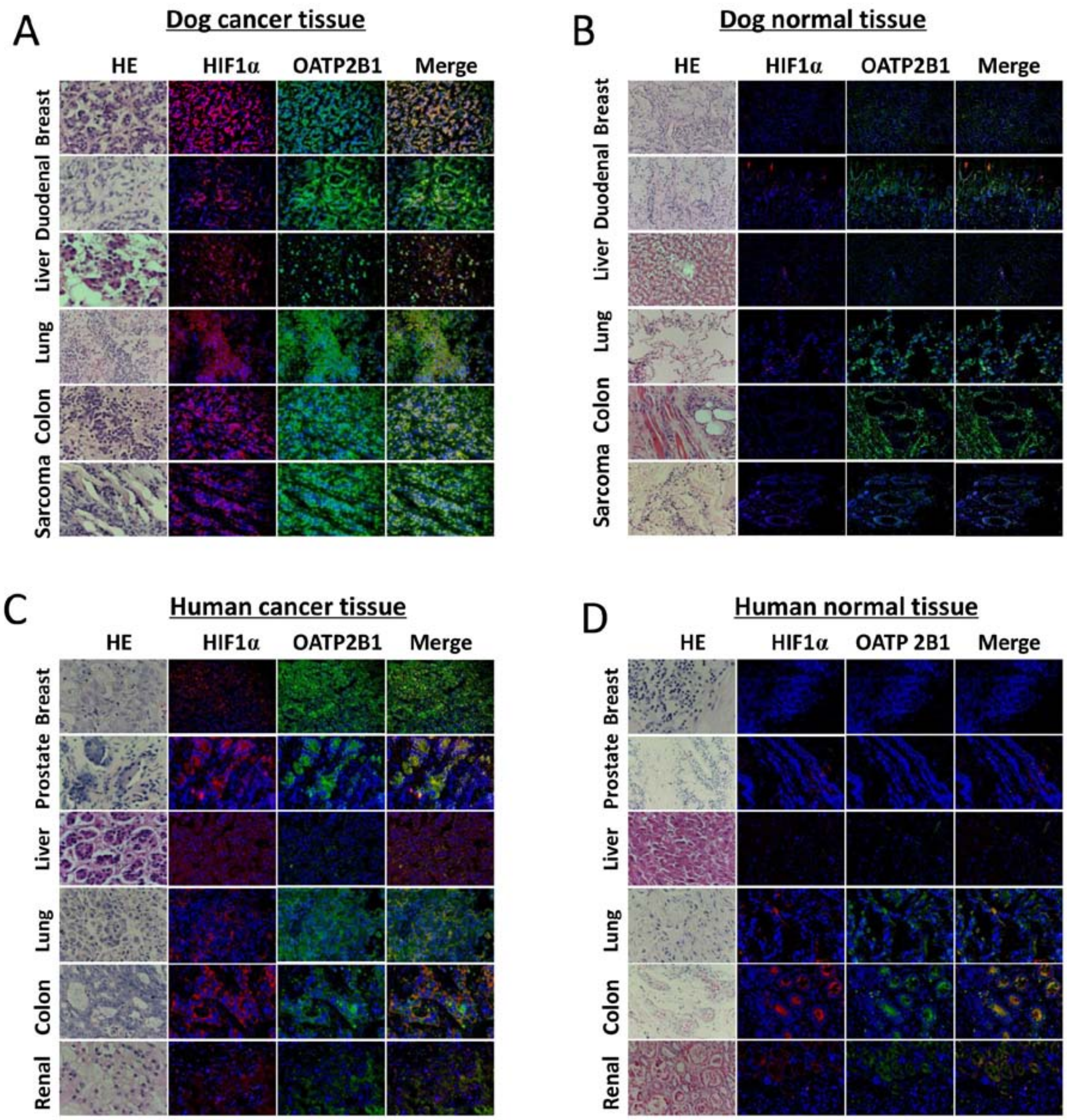

Supplementary Figure S2: mQDL analysis of HIF-1α and OATP2B1 in multiple types of paired cancer and normal tissues of either canine (A and B) or human (C and D) origin.

Supplementary Table S1. Detailed information on dogs bearing spontaneous tumors.

Species	Age (yr)	Sex	Pathologic diagnosis
Beagle	7–8	Female	Breast cancer
Cocker Spaniel	11–12	Female	Multi-organ metastases cancer (including lung, liver, duodenum and thymus)
Pekingese	5–6	Male	Epidermis sarcoma
Pekingese	11–12	Male	Colon cancer
Pomeranian	7–8	Male	Testicular cancer

Supplementary Table S2. Sequences of primers used for qRT-PCR.

Canine *Vegf-a*	F: CTATGGCAGGAGGAGAGCAC
	R: CTATGTGCTGGCCTTGATGA
Canine *Glut1*	F: CCTGCAGTTTGGCTACAACA
	R: CAGGTTCATCATCAGCATGG
Canine *Oatp1b3*	F: TGGTCAGTCAGCATCTGAGG
	R: CAGCAACTGGTTTGCTTTCA
Canine *Oatp2b1*	F: GGGAACACAGCCTTGATTGT
	R: TGTGGGAGACTCATGAGCAG
Canine *Oatp4a1*	F: GGCCAGAAGGTGTACCGAGA
	R: ACCAGAAGGAGGGGCTTTCT
Canine *Oatp5a1*	F: AGGCTCTTGCTGGGAGTACA
	R: GGCATACTTATCGGCGTTGT
Canine *Gapdh*	F: CCCACTCTTCCACCTTCGAC
	R: TGTCATACCAGGAAATGAGCTTGA
Human *VEGF-A*	F: GCTACTGCCATCCAATCGAG
	R: CTCTCCTATGTGCTGGCCTT
Human *Glut1*	F: GTCACCATCCTGGAGCTGTT
	R: GAAGGCCGTGTTGACGATAC
Human *OATP1B3*	F: GGGTGAATGCCCAAGAGATA
	R: ATTGACTGGAAACCCATTGC
Human *OATP2B1*	F: TCAAGCTGTTCGTTCTGTGC
	R: GTGTTCCCCACCTCGTTGAA
Human *OATP4A1*	F: CTGCCAGCCAGAACACTACA
	R: AGAAGGAGGGGCTTTCTCTG
Human *OATP5A1*	F: TGAGCCAGTCTGTGGATCAG
	R: ATCACTTGGCGACTTTGGAC
Human *GAPDH*	F: GACAACAGCCTCAAGATCATCAG
	R: ATGGCATGGACTGTGGTCATGAG
